# Interspecies comparison of probiotics isolated from different animals

**DOI:** 10.14202/vetworld.2018.227-230

**Published:** 2018-02-21

**Authors:** Amr M. Abdou, Riham H. Hedia, Shimaa T. Omara, Mohamed Abd El-Fatah Mahmoud, Mai M. Kandil, M. A. Bakry

**Affiliations:** 1 Department of Microbiology and Immunology, National Research Centre 12622 Dokki, Giza, Egypt; 2 Department of Parasitology and Animal Diseases, Division of Veterinary Research, National Research Centre, 12622 Dokki, Giza, Egypt

**Keywords:** *Lactobacillus*, multiplex polymerase chain reaction, probiotics

## Abstract

**Aim:**

The aim of the current study was to isolate and identify naturally occurring probiotic *Lactobacillus* species in different animals with the different environmental background including fish, and farm animals to investigate interspecies differences in probiotics on the species level.

**Materials and Methods:**

A total of 44 fecal and milk samples were collected under aseptic conditions from cattle, buffalo, camel, sheep, goats, and fish. The samples were cultured, and the isolated strains were confirmed biochemically and molecularly using 16S rRNA multiplex polymerase chain reaction (PCR) analysis following DNA extraction from the bacterial isolates.

**Results:**

A total of 31 isolates identified as lactobacilli were isolated from cattle milk, goat feces, sheep feces, fish feces, buffalo milk, camel milk, and goats’ milk. *Lactobacillus* species were identified based on the size of the PCR product. The results showed that different species were different in their lactobacilli content. At the same time, there were some differences between individuals of the same species.

**Conclusion:**

The diversity of probiotic strains isolated from different animal species implies different types of benefits to the host. Although it would be both money - and time-consuming research, discovering the benefit of each of these strains may provide very important information for the health of both human and animal. Furthermore, transferring these beneficial effects either to individuals within the same species or between different species would be of great importance.

## Introduction

The consumption of some beneficial microorganisms in traditional foods including yogurt, cheese, and milk was associated with protection against diseases and extended lifespan [1,2]. These microorganisms were identified as “probiotics,” and they have become the subject of study that drove attention of many scientists.

Probiotics are defined as the living microorganisms which, when administered in adequate amounts, confer health benefits to the host [3]. Probiotic microorganisms present health-promoting properties, among them the beneficial balance of the intestinal microbiota that can be also associated with other benefits to the host. Probiotics have been prescribed for patients with gastrointestinal disease and complaints [4].

There is a set of cumulative evidence that supports the use of probiotics, both in food products and supplements provide protection against infectious diseases including respiratory infections [5,6]. The most commonly used strains of probiotics are members of Lactobacilli, Enterococci, and Bifidobacteria groups which are families of lactic acid bacteria (LAB). LAB represents a heterogeneous group of microorganisms that are present in the normal diet of many people and also in the gastrointestinal and urogenital tract of animals.

The aim of the current study was to isolate and identify naturally occurring probiotic *Lactobacillus* species in different animals with a different environmental background including fish, poultry, and farm animals to investigate interspecies differences in probiotics on the species level.

## Materials and Methods

### Ethical approval

Institutional Animal Ethics Committee and local laws and regulations were considered in applying our experiment.

### Collection of samples

A total of 44 fecal and milk samples were collected under aseptic conditions from cattle, buffalo, camel, sheep, goats, and fish. The samples were collected in sterile carriers and stored on ice until delivery to the laboratory. Once delivered to the laboratory, they were taken to the procedure for the isolation of probiotic strains.

### Isolation of probiotic strains

*Lactobacillus* spp. as the most commonly used probiotics was isolated from the collected samples using MRS medium as a selective medium. One gram of each fecal sample, as well as 1 ml of each of the milk samples, was dissolved in 100 ml of MRS broth at pH 6.5. After dissolving into MRS broth, they were shaken homogeneously and incubated at 37°C for 24 h in aerobic condition. The cultures were sub cultured at 37°C under low pH (pH 4.5) and anaerobic condition in the presence of 10% CO_2_ to remove unwanted bacteria. After seven subcultures, the bacterial culture was streaked onto MRS agar media at pH 4.8. Finally, a single colony of *Lactobacillus* was selected by observing the colony morphology and some biochemical tests including Gram staining and catalase test. The culture was maintained in MRS broth at pH 5.5 [7].

### Characterization of isolated bacteria

The isolated bacteria were evaluated by different biochemical and molecular tests including Gram stain and catalase test as well as bacterial morphology. The species of promising probiotic LAB strains, as identified by being Gram-positive and catalase-negative, were further characterized by API CHL 50 system (BioMerieux, Marcy L’Etoile, France) and 16S rRNA multiplex polymerase chain reaction (PCR) analysis.

### Gram staining

The culture after 24 h growth was taken on a slide and heat fixed. The smear on the slide was flooded with crystal violet and incubated for 1 min. The slide was then washed in a gentle and direct stream of tap water for 2 s. The slide was again flooded with iodine mordant and incubated for 1 min the slide was washed again in a gentle and direct stream of tap water for 2 s. Later on, counterstain safranin was added, and the slide was then washed with 95% ethanol and observed under the microscope [8].

### Catalase test

Fresh liquid cultures were used for catalase test by dropping 3% hydrogen peroxide solution onto 1 ml of overnight cultures. The isolates, which did not give gas bubbles, were chosen. Since lactobacilli are known to be catalase negative.

### Molecular identification of probiotic strains

Strains were confirmed using 16S rRNA multiplex PCR analysis following DNA extraction from the bacterial isolates as described by Kwon *et al*. [9]. The primers’ sequences and the expected sizes of PCR products are shown in Table-1. A total of 25 μl reaction mixture contained 12.5 µl of EmeraldAmp Max PCR Master Mix (Takara, Japan), 5 µl primer mixture comprising 50 pmol primer, 4.5 µl of water, and 3 µl of DNA template. PCR amplification was performed Applied Biosystem 2720 thermal cycler, and DNA fragments were amplified as follows: Initial heating at 94°C for 2 min, followed by 35 cycles consisting of denaturation at 94°C for 20 s, annealing at 51°C for 40 s, extension at 68°C for 30 s, and a 7 min final extension step at 68°C. Amplicons were separated on 1.5% agarose gel by electrophoresis and analyzed by RedSafe Nucleic Acid Staining Solution (Intron Biotechnology, Korea).

**Table-1:** Multiplex PCR primers that were used in the current study.

Target bacteria	Sequence (50 to 30)	Target site	Product (bp)
All *Lactobacillus*	CCACCTTCCTCCGGTTTGTCA	1178-1198	-
All *Lactobacillus*	AGGGTGAAGTCGTAACAAGTAGCC	1499-1522	-
*L. casei*-group	TGGTCGGCAGAGTAACTGTTGTCG	472-495	727
*L. acidophilus*	AACTATCGCTTACGCTACCACTTTGC	2079-2104	606
*L. delbrueckii*	CTGTGCTACACCTAGAGATAGGTGG	1015-1039	184
*L. gasseri*	ATTTCAAGTTGAGTCTCTCTCTC	1748-1770	272
*L. reuteri*	ACCTGATTGACGATGGATCACCAGT	94-118	1105
*L. plantarum*	CTAGTGGTAACAGTTGATTAAAACTGC	1900-1926	428
*L. rhamnosus*	GCCAACAAGCTATGTGTTCGCTTGC	1922-1946	448

PCR=Polymerase chain reaction

### Long-term preservation of isolates

Identified strains were preserved in MRS broth medium containing 20% (v/v) glycerol as frozen stocks at −80°C. The glycerol stocks of samples were prepared by mixing 0.5 ml of active cultures and 0.5 ml MRS medium including 40% sterile glycerol.

## Results

Lactobacilli isolated from fecal and milk samples of different animal species including cattle, buffalo, camel, sheep, goats, and fish were subjected to characterization and identification using different biochemical and molecular identification methods. Lactobacilli were identified as Gram-positive and catalase-negative bacilli. *Lactobacillus* strains were further identified either by API 50 CHL systems or 16S rRNA multiplex PCR analysis.

### Multiplex PCR analysis

**Figure-1:**
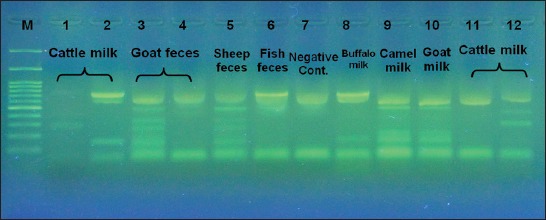
Agarose gel electrophoreses of polymerase chain reaction (PCR) products from multiplex PCR assays. Multiplex PCR assays were performed with a mixture of seven species-specific or group-specific primers for *L. acidophilus*, *L. bulgaricus* (same as *L. delbrueckii* subsp. *bulgaricus*), *L. casei*-group *L. gasseri*, *L. plantarum*, *L. reuteri*, and *L. rhamnosus* and two bacterial conserved primers. Lanes 1-12 designate the PCR product from each genomic DNA extracted from single or mixed cell suspension isolated from representative host used as PCR template. Lane 1: *L. casei*, *L. gasseri*; Lane 2: *L. casei*, *L. acidophilus, L. delbrueckii*; Lane 3: *L. acidophilus, L. rhamnosus, L. plantarum, L. gasseri, L. delbrueckii;* Lane 4: *L. acidophilus;* Lane 5: *L. rhamnosus, L. plantarum, L. gasseri*, *L. delbrueckii*; Lane 6: *L. casei*; Lane 7: *Negative control*; Lane 8: *L. casei*, *L. acidophilus, L. delbrueckii*; Lane 9: *L. acidophilus, L. rhamnosus, L. gasseri, L. delbrueckii*; Lane 10: *L. acidophilus, L. rhamnosus, L. gasseri, L. delbrueckii*; Lane 11: *L. acidophilus*, Lane 12: *L. acidophilus, L. rhamnosus*, *L. plantarum*; Lane M, 100 bp-DNA ladder.

The results of 16S rRNA multiplex PCR analysis are showed in Figure-1. A total of 31 isolates identified as lactobacilli were isolated from cattle milk, goat feces, sheep feces, fish feces, buffalo milk, camel milk, and goat milk. *Lactobacillus* species were identified based on the size of the PCR product [9]. The results showed that different species were different in their lactobacilli content. On the same time, there were some differences between individuals of the same species. At the other hand, the results showed some similarities on species level as shown by the lactobacilli contents of fecal samples derived from goat and sheep as well as milk samples derived from cattle and buffalo. The same was true also for milk samples derived from goat and camel.

## Discussion

There is a growing interest in identifying the health benefits of probiotics when consumed in adequate amounts. The health-promoting properties of probiotics include their immunoregulatory effects as well as their beneficial balance on the intestinal microbiota. The immunoregulatory effect is suggested to occur through the generation of regulatory T-cells and the generation of IL-10 [10]. Gastrointestinal infections cause imbalance or dysbiosis of the gut microbiota which is associated with many diseases [11]. The presence of probiotics in the gastrointestinal tract is very important to neutralize the harmful bacteria and restore the balance of the intestinal microbiota [12,13].

Probiotics are living microorganisms which, when administered in adequate amounts, confer health benefits to the host [3]. Several genera of bacteria and yeast have been proposed as probiotic cultures. The most commonly used strains of probiotics are members of Lactobacilli, Enterococci, and Bifidobacteria groups. LAB represents a heterogeneous group of microorganisms that are present in the normal diet of many people and also in the gastrointestinal and urogenital tract of animals, and some of these claimed to be probiotics. Probiotics belonging to the genus *Lactobacillus* have been isolated from a variety of habitats, including plant and dairy products, meat products, sewage and manure, and humans and animals. Due to the fact that the beneficial effects of probiotics can vary between strains, the selection of the most suitable ones will be crucial for their use in the prevention or treatment of specific diseases.

Interspecies diversity of probiotic microorganisms is the result of several factors including nutrition, infections, antibiotics, stress, and various disease conditions. Specific probiotics have been suggested to be effective in alleviating the duration and severity of acute rotavirus gastroenteritis [14,15]. In addition, probiotics are able to reduce the risk of respiratory tract infections [7,16] which in most cases are of viral origin. Probiotics are likely to have an impact through gut mucosa by balancing the local microbiota [17], by inhibiting the growth of pathogenic microorganisms [18], and by enhancing local and systemic immune responses [19]. They may also influence the composition and activity of microbiota in the intestinal contents.

In the current study, 16S rRNA multiplex PCR analysis was used to identify and compare probiotic strains in some animal species from different habitats including fish and farm animals. We were able to isolate and identify different *Lactobacillus* strains from different animal hosts. The results showed that lactobacilli diversity were not only on the species level but also on the individual level as different lactobacilli were identified within the same species. This diversity could be due to environmental factors as well as dietary factors. On the other hand, the results showed some similarities on species level as represented with the similarity between the close relatives goat and sheep in their fecal samples as well as the similarity between cattle and buffalo in their milk samples. The same was true also for goat and camel in their milk samples as indicated by their lactobacilli contents. Further studies are needed to investigate the possible beneficial effect of some of the isolated strains as a potential treatment for some diseases.

## Conclusion

The diversity of probiotic strains isolated from different animal species implies different types of benefits to the host. Although it would be both money - and time-consuming research, discovering the benefit of each of these strains may provide very important information for the health of both human and animal. Furthermore, transferring these beneficial effects either to individuals within the same species or between different species would be of great importance.

## Authors’ Contributions

AMA, RHH, STO, MAEM, MMK, and MAB conceived the study, carried out the laboratory work, and analyzed the data. All authors performed the fieldwork and collected the samples. AMA and MAEM drafted the manuscript. All authors read and approved the final manuscript.
